# Congestion, decongestion, renal function, and diuretics in (ESC) heart failure—what’s new?

**DOI:** 10.1093/eschf/xvag054

**Published:** 2026-02-13

**Authors:** Jan Biegus, Robert Zymlinski, Piotr Ponikowski

**Affiliations:** Department of Cardiology, Clinical Department of Intensive Cardiac Care, Wroclaw Medical University, Faculty of Medicine, Institute of Heart Diseases, Borowska 213, Wroclaw 50-556, Poland; Department of Cardiology, Clinical Department of Intensive Cardiac Care, Wroclaw Medical University, Faculty of Medicine, Institute of Heart Diseases, Borowska 213, Wroclaw 50-556, Poland; Department of Cardiology, Clinical Department of Intensive Cardiac Care, Wroclaw Medical University, Faculty of Medicine, Institute of Heart Diseases, Borowska 213, Wroclaw 50-556, Poland

**Keywords:** Congestion, Decongestion, Diuretics, Renal function, Biomarkers

## Introduction

In 2024, we launched a virtual issue in *ESC Heart Failure* focusing on congestion and decongestion pathophysiology, renal function, and diuretic response in heart failure (HF).^1^ The virtual issue is intended to serve as both a comprehensive resource of already published articles in the Journal and an invitation for authors to contribute their high-quality research and insights to advance the field further.

Here, we will highlight some key points from several interesting, recent papers from ESC Heart Failure on this topic.

Proper assessment of congestion is a key clinical skill upon which diagnostic and therapeutic decisions are based in everyday practice, underscoring its importance and clinical relevance. Although this assessment is performed daily, it often remains challenging—partly because of congestion heterogeneity (intravascular vs tissue congestion) and the fact that there are no simple, objective measures that clearly define this phenomenon (analogous to the need for objective markers of diuretic response in HF).^2,3^ Therefore, the development of techniques, modalities, and biomarkers that enable objective evaluation of congestion represents an important area for scientific research in HF.

Pulmonary congestion was a topic of several interesting recent papers. In the first study, the authors prospectively compared the diagnostic utility of low-dose chest CT and chest radiographs in patients with dyspnoea and suspected acute HF (AHF).^4^ In simple terms, the odds of diagnosing adjudicated AHF and echo-BNP confirmed AHF were up to four times greater using CT vs chest X-ray. The authors concluded that low-dose chest CT outperformed chest radiographs in detecting pulmonary congestion. As the primary imaging method, CT detects one more AHF in every 12.5 patients and avoids one false-positive diagnosis in 20 patients. The other group examined the clinical utility of remote dielectric sensing (ReDS) (in a blinded manner) to quantify pulmonary congestion at admission and discharge.^5^ The ReDS ratio was independently associated with all-cause mortality and HF-related re-hospitalizations (the study’s primary endpoint).^5^ In the secondary analysis of the OptiLink HF trial, the remote monitoring of thoracic impedance as a marker reflecting pulmonary congestion was examined in HF patients with concomitant chronic kidney disease (CKD), defined as eGFR <60 mL/min/1.73 m.^2^ The presence of CKD was associated with a higher incidence of fluid status alerts and a higher risk of the primary outcome (the composite of all-cause death or first CV hospitalization).^6^ Surprisingly, the management of the alerts (according to the guidelines) was associated with lower primary outcome events in patients without CKD, but not in the CKD group.^6^ Guazzi *et al*. have provided an in-depth, comprehensive review of the biomarkers of lung congestion/hypoperfusion and injury in AHF.^7^ This paper provides a new perspective on our understanding of the pathophysiology of lung dysfunction and respiratory failure beyond the HF population. The authors provided extensive data on several aspects of lung function, injury, and the metrics that can be used to reflect lung health/damage in HF, including pulmonary surfactant proteins (A, B, C, and D), sST2, ADM, Ca125, CD146, GDF-15, and adrenomedullin.^7^

The latter biomarker (pro-adrenomedullin) was measured at admission and discharge of 233 unselected AHF patients. In this observational, prospective, single-centre study, pro-adrenomedullin concentrations were significantly associated with congestion at both timepoints. The biomarker was also associated with in-hospital worsening of HF, while discharge pro-adrenomedullin was associated with post-discharge death.^8^ The other congestion biomarker, Ca125, was recently assessed in the HFpEF population, where it was shown to be elevated and strongly, independently associated with the risk of HF hospitalization.^9^

In another study that analysed the data from the SWEDEHEART registry (*n* = 87,177), the development of left ventricular (LV) dysfunction and/or congestion after the myocardial infarction was present in 13%–32% of cases. Notably, profiles with LV dysfunction and/or congestion were linked to a 2–3 times greater risk of subsequent death or HF admission during the 3-year follow-up.^10^ The impact of fluid status and risk of intravascular congestion after the cardiovascular insult related to myocardial infarction was also assessed in a study by Nogi *et al*.^11^ The authors used estimated plasma volume status (calculated from haematocrit and haemoglobin using the Strauss-derived Duarte formula) and found that discharge plasma volume was independently associated with high post-discharge all-cause mortality.^11,12^ Both studies clearly highlight that the development of congestion after acute coronary syndrome is associated with poor outcomes, and efforts should be made to effectively identify these patients.

Cardio-renal syndrome in HF represents the complex interplay between the failing heart and the kidney. In the *post hoc* analyses of EMPA-REG OUTCOM, Sharma *et al*. examined the bidirectional relationship between kidney function and cardiovascular (CV) events in patients with type 2 diabetes and CV disease.^13^ In placebo-treated participants of the study, the authors report the bidirectional associations between kidney events and subsequent CV events, and CV events (only HF hospitalizations), and increased risk of subsequent kidney events.^13^ Importantly, aside from HF hospitalizations, CV events like non-fatal stroke, non-fatal myocardial infarction, and CV death were not linked to kidney events.^13^ In the meta-analysis of 37 studies, a total of 3 533 583 patients with HF were included, of whom 774 887 had acute kidney injury (AKI), with a pooled incidence of 33% (95% CI: 32–35%).^14^ The incidence of AKI was higher in AHF compared to the chronic HF population.^14^ AKI was associated with a higher risk of both in-hospital and out-of-hospital mortality. Several clinical and laboratory variables were identified as predictors of AKI development in this huge cohort, such as diabetes mellitus, NTproBNP, blood urea nitrogen >24 mg/dl, and serum albumin.^14^ In another, smaller study, the predictors of worsening renal function (WRF) were: hyponatremia, haemoglobin concentration, white blood cell count, and prior diuretic use.^15^

Watanabe *et al*. added very interesting mechanistic insights into the prognostic information related to the so-called true WRF. The authors classified the cohort of 1103 AHF patients based on the presence of WRF and fractional excretion of urea nitrogen (FeUN) at discharge into categories: low ≤32.1%; medium >32.1% and ≤38.0%; and high >38.0%.^16^ The primary outcome of this study was HF readmission. Interestingly, in multivariable analysis, WRF was associated with increased risk of the primary outcome, but the risk was significantly higher in patients with WRF and either low or high FeUN. In contrast, patients in the group with medium FeUN had the lowest risk of subsequent AHF events.^16^ In the earlier study, the same group demonstrated that FeUN may be a potential marker of volume status in HF.^17^ The authors suggested that a low FeUN value (FEUN ≤32.1) may represent intravascular dehydration, whereas a high FeUN value (FEUN >38.0) may reflect residual congestion; and both may be associated with poor outcomes.^17^

Spot urine sodium has been shown to be a marker of loop diuretic response, and a reliable biomarker that reflects neurohormonal drive and decongestive capabilities in AHF.^18,19^ The ESC Guidelines and expert documents recommend the post-diuretic spot urine sodium as the best available marker to assess diuretic response. Iwanek *et al*. demonstrated that although the spot urine sodium <70 mmol/l is associated with high accuracy in detecting an insufficient diuretic response (< 600 ml within the first 6 h after intravenous administration of diuretic), with a sensitivity of 0.78 and a specificity of 0.89, the urine sodium adjusted for urine dilution (urine creatinine) performed better than urine sodium alone. The urine sodium to urine creatinine ratio (uNa+/uCr <1.675 mmol/mg) had a sensitivity of 0.83 and a specificity of 0.97, with an AUC of 0.956 (0.915–0.997), *P* < 0.001.^20^ In conclusion, incorporating urine sodium normalized to urine creatinine substantially improves the accuracy of early diuretic response assessment, providing a more robust and physiologically grounded biomarker of decongestive efficacy in AHF.

## Data Availability

This article did not involve the generation or analysis of any original data. Therefore, no data is available for sharing.
